# “Mind the Trap”: Mindfulness Practice Reduces Cognitive Rigidity

**DOI:** 10.1371/journal.pone.0036206

**Published:** 2012-05-15

**Authors:** Jonathan Greenberg, Keren Reiner, Nachshon Meiran

**Affiliations:** Department of Psychology, Ben-Gurion University of the Negev, Beer-Sheva, Israel; Institute of Psychiatry at the Federal University of Rio de Janeiro, Brazil

**Keywords:** *Keywords:* Mindfulness Meditation, Cognitive Rigidity, Einstellung, Water-Jar Task

## Abstract

Two experiments examined the relation between mindfulness practice and cognitive rigidity by using a variation of the Einstellung water jar task. Participants were required to use three hypothetical jars to obtain a specific amount of water. Initial problems were solvable by the same complex formula, but in later problems (“critical” or “trap” problems) solving was possible by an additional much simpler formula. A rigidity score was compiled through perseverance of the complex formula. In Experiment 1, experienced mindfulness meditators received significantly lower rigidity scores than non-meditators who had registered for their first meditation retreat. Similar results were obtained in randomized controlled Experiment 2 comparing non-meditators who underwent an eight meeting mindfulness program with a waiting list group. The authors conclude that mindfulness meditation reduces cognitive rigidity via the tendency to be “blinded” by experience. Results are discussed in light of the benefits of mindfulness practice regarding a reduced tendency to overlook novel and adaptive ways of responding due to past experience, both in and out of the clinical setting.

## Introduction

Experience may blind us from recognizing obvious solutions to problems. Research shows that physicians and health care professionals are likely to overlook the correct diagnosis in cases which do not match their experience [1]. Similar findings have been reported concerning difficulties in reframing clinical situations as experienced by healthcare professionals [2], [3], and difficulties of managers and decision makers in replacing existing procedures with new, improved and simpler ones [4]. This “blinding” to novel solutions may be considered a form of cognitive rigidity, which has commonly been defined as a resistance to change in beliefs, attitudes or personal habits [5], or the tendency to develop and perseverate in the use of mental or behavioral sets [6].

Such cognitive rigidity may play a key role in psychopathlogy (for reviews see [6], [7], see also [8]). It has been closely linked to the inability of suicidal individuals to consider alternatives that may be accessible to another person [9], [10], as well as to rumination, a major risk factor of depression [11]. Similar forms of cognitive rigidity were also indicated in obsessions [12], [13], alcohol dependence [14], eating disorders [15], and Attention Deficit Disorder [16]–[20]. In this paper, we propose that mindfulness meditation may provide a means of decreasing the aforementioned type of cognitive rigidity.

Mindfulness is a term which has developed from early eastern traditions and has been commonly defined as "paying attention in a particular way: on purpose, in the present moment and non-judgmentally” [21]. Although some have directly linked mindfulness to a practice of meditation (e.g. [22]–[24]), others (e.g. [25]–[27]) have referred to it as rather independent from meditation practice. Mindfulness has additionally been described as a theoretical construct, a psychological process [28], and a trait (see [29], [30] for a recent discussion of discrepancies between various definitions of mindfulness). Nevertheless, mindfulness has been commonly claimed to involve regulation of the focus of attention towards the current experience, a willingness to come in contact with and be receptive to experience rather than avoid it or cope by means of repression, and to involve adopting a “beginners mind” and seeing things in a “fresh” way [31]. These last attributes of mindfulness in particular seem to potentially immune one from being blinded by experience.

Mindfulness has received a great deal of empirical attention over the last three decades, and various psychotherapeutic techniques based on mindfulness have been developed (e.g. [22], [32], [33]). Mindfulness based interventions have been shown to alleviate symptoms of a variety of clinical conditions such as suicidal ideation and manic symptoms [34], relapse reduction in recurrent major depression (see [35]–[37] for recent reviews), rumination ([38], [39] see [40] for differential effects of mindfulness on adaptive and maladaptive rumination), addictions and substance use disorders [41], [42], eating disorders (see [43] for a review), generalized anxiety [44], obsessive compulsive disorder (OCD) [32], and attention deficit/hyperactivity disorder (ADHD) [45]. Interestingly, many of the disorders which benefit from mindfulness mediation are also characterized by some form of rigidity, suggesting that the efficacy of mindfulness may perhaps be mediated by reduced rigidity.

In addition to studying mindfulness as a form of therapeutic intervention, there has been a growing body of research over the last years examining various cognitive abilities related to mindfulness, most of which focusing on various measures of attention and memory (see [46] for a review). Only few studies have directly addressed the relation between mindfulness and cognitive flexibility or rigidity. Although some studies did not find differences between meditators and non-meditators in rigidity related tasks (e.g [47], [48]), others have found that meditators exhibit decreased Stroop interference [49], [50](in a Zen meditation sample). The Stroop task requires participants to name the ink color in which color words are written. The interference reflects automaticity with regards to the fact that participants cannot avoid reading the words. This inability to flexibly adapt to novel and non-habitual task requirements may be taken as evidence for inflexibility. Along the same line, other studies found that meditators exhibit superior visual perspective switching on a multiple perspective images task [51], exhibit superior verbal fluency [52], [53], and perform better than controls on a category production task [54] and the Hayling task, requiring participants to complete sentences with unrelated and nonsensical words [53]. Mindfulness meditators have also been shown to exhibit reduced rumination compared to controls [38]–[40], which may also be related to reduced rigidity as reflected in the adoption of repetitive thought patterns concerning distressing symptoms, their causes and implications [55].

Importantly, none of the aforementioned tasks tap the tendency to be “blinded” by experience, and overlook simple, obvious novel solutions to a given problem, which is what we studied in this work. To this end, we adopted the water jar paradigm developed by Luchins [56]. We had chosen this task over other measures of rigidity since it directly captures the notion of missing obvious adaptive solutions that lie right “under the nose” due to being caught up in learned and repetitive thought patterns. Furthermore, this particular form of rigidity seems most relevant to mindfulness meditation, which is said to involve relating to the present situation with decreased reliance on former knowledge and experience [57]. The water jar paradigm was designed to measure the *Einstulling effect*, a term used to describe rigid thought patterns formed through experience which prevents identifying more adaptive approaches and solutions. In this task, participants are required to use three hypothetical jars to obtain a specific amount of water. Initial problems are solvable by the same complex formula, but in later “critical” problems a much simpler formula is also appropriate. In these trials, experience is said to comprise a “trap” which may result in overlooking the simple formula. A rigidity score is compiled, reflecting the degree of perseverative use of the complex formula. Since mindfulness is said to be characterized by focusing on the present moment with a “beginners’ mind”, we hypothesize that mindfulness experience would result in lower rigidity scores. This hypothesis was examined in two studies. Experiment 1 compared a sample of experienced mindfulness meditators with a comparison group of people who had taken an active interest in mindfulness and had registered to a mindfulness retreat, yet at the time of assessment did not have any formal meditation experience. We chose this group in an attempt to match meditators inclinations and personality characteristics. In Experiment 2, we compared two, randomly assigned groups of non-meditators: a group who underwent eight sessions of structured mindfulness training and a waiting list group, before and after mindfulness training of the mindfulness group.

## Methods

### Experiment 1

#### Participants

The experienced meditation group was composed of 14 mindfulness (Vipassana, [29]) meditators (7 males, mean age = 37.29, *SD* = 8.44), having minimum meditation experience of three years (*M* = 8.54 years, *SD* = 4.39) and practicing regularly (*M* = 3.20 hours per week, *SD* = 2.26). Data regarding experience were available for all but two experienced meditators. The control group, from here on referred to as the Pre-meditation group, was composed of 21 individuals (8 males, mean age = 31.24, *SD* = 10.29) with no meditation experience, who had registered for their first meditation retreat. Participants were recruited via poster ads and telephone through a mindfulness meditation (Vipassana) association in Israel. There was no significant difference between groups in age [*t*(25) = 0.88, ns] nor in gender (*p* = .36, Fisher’s exact test). Self reported Psychometric Entrance Test (PET) score, the Israeli equivalent of the SAT scores, served to assess the equivalence of the groups in academic abilities. PET scores have previously been found to highly correlate (r = 0.81) with scores on the Wechsler Adult Intelligence Scale-Revised [58]. A PET equivalent score was calculated for two participants who had not taken the PET, via a regression model provided by Oren (Personal communication 6.11.2010) for the average high school matriculation exam score, based on a sample of 65,000 students studying in academic institutions, data of which can be found at the Israeli National Institute for Testing and Education (NITE). Six participants from the pre-meditators group and two from the meditators group were unable to report PET or matriculation exam data. Meditators (*M* = 656.91, *SD* = 52.79) did not significantly differ in their academic abilities as assessed by the PET score from pre-meditators [*M* = 689.27, *SD* = 68.37; *t*(23) = 1.34, ns]. All participants were offered a mindfulness related book as compensation for attendance. The experiment had received approval from the psychology department’s ethics committee in Ben-Gurion University.

#### Measures

Water jar task: A computerized version of the task was administered using E-Prime (Psychological Software Tools, Inc.), with problems adapted both from Luchins [56] and Schultz and Searman [59] (see Table 1). Participants viewed three jars onscreen marked A, B, and C with numbers indicating their size, and a target cup indicating the goal to obtain. Participants were instructed to obtain the goal amount of water by adding or subtracting the jars given in each problem, while applying the simplest and shortest solution.

**Table 1:** Water Jar Problems in Both Studies.

	Experiment 1				Experiment 2				
Trial Type	Jar A	Jar B	Jar C	Goal toobtain	Jar A	Jar B	Jar C	Goal toobtain	Shortest Solution
Example	29	3	0	20	29	3	0	20	A-3B
Set	31	61	12	6	31	61	12	6	B-A-2C
Set	22	57	10	15	22	57	10	15	B-A-2C
Set	18	59	16	9	18	59	16	9	B-A-2C
Set	20	67	13	21	20	67	13	21	B-A-2C
Set	22	57	10	15	22	57	10	15	B-A-2C
Set	24	52	3	22	21	127	3	100	B-A-2C
Set	19	42	3	17	18	43	10	5	B-A-2C
Set	21	127	3	100	24	52	3	22	B-A-2C
Set	18	43	10	5	19	42	3	17	B-A-2C
Set	14	163	25	99	14	163	25	99	B-A-2C
Critical	18	48	4	22	18	48	4	22	A+C
Critical	15	39	3	18	15	39	3	18	A+C
Critical	23	49	3	20	23	49	3	20	A−C
Critical	–	–	–	–	7	16	2	5	A−C
Extinction	14	39	8	6	14	39	8	6	A−C
Extinction	13	37	5	18	13	37	5	18	A+C

**Figure 1:**
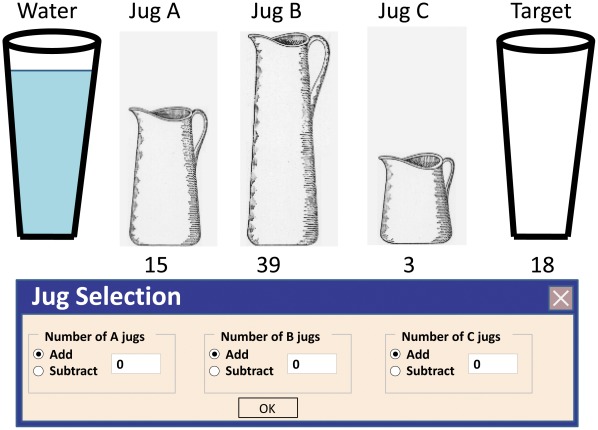
Illustration of computer display on critical trial (English version). Following instructions to apply the shortest and simplest solution, participants entered the desired number of jugs of each type in the dialog box at the bottom, and toggled between the “add” and “subtract” options to reach the target amount specified on the right.

Participants inserted their solution by toggling between the “add” and “subtract” options and typing the number of jars desired to be added or subtracted in the adjacent textbox beneath each jar (see Figure 1 for an example translated from Hebrew to English). Participants were provided with scrap paper and a pen to assist in calculations. After verifying that participants comprehended instructions and mastered onscreen navigation, participants were given an example question and were encouraged to ask questions. Once solved correctly, the experimenter left the room and participants independently solved the presented problems. The first trials were *set trials,* solvable by the formula B-A-2C, in which participants were required to add one B jar, subtract one A jar, and subtract 2 C jars (e.g. obtaining 100 units of water with jars the capacity of 21, 127, and 3 units by performing 127−21−3−3 = 100). Once 6 out of the maximum of 10 set trials were correctly solved, participants were presented with 3 *critical trials*, solvable both by the complex B-A-2C formula and by a simple formula: either A+C or A-C (e.g. obtaining 18 units of water with jars the capacity of 15, 39, and 3 units by performing 15+3 = 18, as opposed to using the more complex formula - 39−15−3−3 = 18). Participants were then presented with two *extinction trials*, solvable only with the simple formula. Participants were instructed not to spend more than 5 minutes on each problem. One rigidity point was given for each critical or extinction trial solved using the complex formula, and for each extinction trial exceeding a cutoff of 60 seconds solving time.

Exclusion from analyses criteria included the use of fractions rather than whole jar numbers, correctly utilizing the ascribed complex solution on less than six “set” trials, and performing any calculation errors or applying alternative novel solutions (which were evidently possible in a few of the problems) on the two final “set” trials prior to critical trials, as well as on critical trials. These were assured in order to facilitate and standardize both mental set formation prior to critical trials, and rigidity score compilation.

#### Procedure

Participants entered a room containing a computer. They signed an informed consent form, and then carried out the water jar task. At the end of the experiment participants had answered a few demographic questions regarding age, psychometric exam score, and meditation experience. At the end of the session participants were debriefed.

### Experiment 2

#### Participants

Seventy six individuals with no former meditation experience were recruited via poster ads hung around Ben-Gurion University campus and email ads sent to all university students, offering a free mindfulness program for those participating in two experimental sessions. The program was due to start in two possible dates several months apart. Exclusion criteria included people with learning disabilities and non-native Hebrew speakers, due to the mathematical and lingual nature of the tasks, as well as people with previous meditation background. Following the first experimental session, participants were randomly assigned to a Mindfulness meditation group (N = 38) intended to partake in the first program and a waiting list (control) group (N = 38) intended to partake in the second program. No significant differences were found between groups in Age [*M* = 25.45, *SD* = 2.56 for meditators, *M* = 26, *SD* = 2.5 for controls, *t*(74) = 0.95, ns], gender (13 male meditators, 15 male controls, *p* = .41, Fisher’s exact test), nor in academic abilities as measured by PET scores [*M* = 662.97, *SD = *62.22 for meditators, *M* = 672.66, *SD* = 57.37 for controls, t(74) = 0.70, ns]. Demographic characteristics of participants included in baseline analysis are depicted in Table 2.

**Table 2:** Demographic characteristics of participants in Experiment 2.

	Controls					Meditators				
		Mean	SD	Min	Max		Mean	SD	Min	Max
Gender	69% Women					69% Women				
Age		25.50	2.74	20	35		26.06	2.63	20	33
EducationLevel	88% under-grad			Under-grad	PhD	88% under-grad			Under-grad	PhD
PET Score		674.43	57.61	567	783		660.38	654.79	480	763
Rigidity scoreat session 1		3.50	2.00	0	6		2.78	2.09	0	6

Data consists of the 32 participants in each group with valid rigidity scores in Session 1.

#### Measures

Alphabet-maze: Since the water jar task may lose much of its efficacy if administered in both experimental sessions (due to previous exposure to short solutions on extinction trials), it had been administered only in the second session, after mindfulness training of the mindfulness group. Initial differences in Einstellung rigidity between groups had been assessed in the first experimental session using a task similar in structure to the water jar task, the Alphabet-Maze task, which is described in detail by Cowen [60]. A Hebrew version of this task was administered via a computer using E-Prime software. An array of Hebrew letters appeared onscreen. Participants were told that the object of the task is to move from upper-left corner of the maze to the bottom-right corner, spelling out words as they go along (the original direction of the English task has been reversed since Hebrew is written from right to left). Once a word has been spelled, participants may move in any direction (up, down, right, left or diagonally) to spell the following word. Words must be at least 3 letters long. As with the water jar task, instructions encouraged solving rather quickly, yet strongly emphasized that the goal was to reach the end via the shortest and most direct solution, using as few letters as possible. After solving two example questions and verifying comprehension of instructions, participants were presented with six *set trials*, solvable by a long solution (a down-right diagonal movement, followed by a straight down movement, and finally directly to the right). Once solved, participants had written on paper the words taking them from start to end, and pressed the space bar to continue to the following trial. The next four trials were *critical* (also named *trap* or *crucial trials*), solvable by both the long solution and a short solution (a straight diagonal path for the upper-left to the lower-right corners). Finally, two *extinction trials* were presented, solvable only by using the short solution. Rigidity scoring was identical to that of the water jar task, i.e. one rigidity point given for each critical or extinction trial solved using the complex formula, and for each extinction trial exceeding a solving time of 60 seconds. A few problems were evidently solvable by more than one solution. Data were considered valid if at least 4 out of the 6 set trials were solved in the intended method, and no alternative solutions were applied in critical trials nor in the two final set trials.

Water jar task: Task was similar to that described in Experiment 1, with a few modifications. Task programming was modified to prevent the possibility of continuing to the next trial if the current one was mathematically incorrect, and order of a few set trials was modified to postpone trials more prone to be given alternative solutions. Both modifications were applied in order to maximize mental set constitution and minimize invalid or unscorable data by preventing the application of mathematically incorrect solutions and minimizing as much as possible the number of solutions other than the intended ones. Additionally, number of critical trials was increased from 3 to 4 (See Table 1) in order to enhance the reliability and hence the statistical power.

#### Mindfulness Program

The mindfulness program was developed in Be’er-Sheva’s Mental Health Center, based on the Mindfulness Based Cognitive Therapy program [33] with adaptations to include handling general stress and everyday difficulty rather than contents limited to depression. The program was conducted over a period of six weeks, and consisted of seven 2 hour group sessions, and an additional half day retreat at the end of the fifth week. During each session the instructor guided the participants through different meditations including breathing meditation, body scan, open awareness meditation, walking meditation and compassion meditation. A detailed description of the instructions provided for the different meditations is presented in Table 3. Each session additionally included different awareness exercises, stories and group discussions to allow a broader understanding of mindfulness principles, and provide the participants with opportunities to share their meditation experiences. Daily home practice of formal meditation of at least 20 minutes was required, as well as informal daily mindfulness practice in which participants attend to regular daily activities in a mindful manner. Audio CD’s with meditation instructions were provided to facilitate home practice. Additionally, participants received a daily email notification directing them to an online diary for filling a report of their daily home practice. During the half day retreat participants practiced different formal meditations in silence, with no exercises or group discussions. The program was led by one of the authors (KR), a trained mindfulness based therapy instructor and psychologist, with a personal mindfulness meditation experience of over ten years. The instructor was blind to the hypothesis of the experiment.

**Table 3:** Instructions for the various meditations in the mindfulness program.

Instructions	Meditation
Participants were asked to sit still with their eyes closed and focus their attention on the sensations of their breath. Whenever theynoticed that their attention wandered off from their breath they were asked to observe the object of their attentionin the present moment, whether it is a thought, an emotion or a sensation, without judgmentor reaction, and then bring their attention gently back to the breath.	Breathing meditation
Participants were asked to sit still with their eyes closed and focus their attention on bodily sensations in different parts of their body, according to the given instructions. When they noticed that their attention had wandered off from the sensations in the specifiedbody part they were asked to observe the object of their attention in the present moment without judgment or reaction, and thenbring their attention gently back to the sensation in the specified body part.	Body scan meditation
Participants were asked to sit still with their eyes closed and focus their attention on different qualities of the present moment,for example, sounds, smells, body sensations and the breath. When they noticed that their attention had wandered off from thepresent they were asked to observe the object of their attention without judgment or reaction, and then bring their attentiongently back to the present moment.	Open awareness meditation
Participants were asked to sit still with their eyes closed and think of someone they care about. Participants were then asked to wishthat this person will be free from suffering, distress or loss, and will experience joy, peace and love. Participants were asked to dothe same for someone they did not know well, for someone with whom they have a conflict, and for themselves.	Compassion meditation
Participants were asked to walk slowly and silently, and notice the different segments and sensations of walking. When they noticedthat their attention had wandered off from walking, they are asked to stand still and notice where their attention is at thepresent moment, without judgment or reaction and then bring their attention gently back to the present momentand resume the walking.	Walking meditation

#### Procedure

In the beginning of each of the two experimental sessions, participants signed an informed consent form. In Session 1 they then performed the Alphabet-maze task. Participants were then randomly assigned to groups. The randomization procedure involved using the “random” function in Python programming language to assign half of the participants to each group by participant number. Session 2 took place near the end of the mindfulness program. Participants completed a Competitor Rule Suppression task (CRS) [61], which does not involve Einstellung rigidity and will not be discussed in the current paper. They then completed the water jar task.

## Results

### Experiment 1

Six participants of the Pre-meditation group and two participants from the experienced meditation group met exclusion criteria and were excluded from the main analysis. Due to our directional hypothesis concerning rigidity scores, we had performed one-tailed tests on both studies, although almost all differences reach a two-tailed significance level. As hypothesized, experienced mindfulness meditators (*M = *1.17, *SD* = 1.75) attained significantly lower rigidity scores than pre-meditators [*M* = 2.93, *SD* = 2.02; *t*(25) = 2.40, *p* = .01, 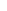 ^2^ = 0.19; Post hoc power, calculated by GPower software [62] = 0.78]. The effect increased considerably once Age and PET scores had been added as a Covariates to the analysis in a one way ANOVA [*F*(1,16) = 9.81, *p*<.01, 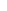 ^2^ = 0.38; Post hoc power = 0.96]. Group differences in rigidity scores remained significant after we had performed the comparison on all 35 original participants (14 experienced meditators, 21 pre-meditators), including those who had met the exclusion from analyses criteria as specified in the Method section, in order to insure that group differences were not accounted for by the exclusion criteria [*t*(33) = 2.42, *p* = .01, 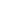 ^2^ = 0.15; Post hoc power = 0.77].

Since there was a certain degree of arbitrariness in determining the time cutoff for extinction trials in calculating rigidity scores, we re-calculated rigidity scores using various alternative solving time cutoffs for extinction trials (60, 90, 120 seconds). The significant group differences in rigidity scores remained in all t-tests (minimal *t* = 2.40, *p* = .01, 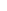 ^2^ = 0.15; Post hoc power = 0.67).

### Experiment 2

Since correlation between rigidity scores of the alphabet-maze and the water jar task was low and non-significant (r = .10, ns) results from these tasks are hereby reported separately.

#### Alphabet-maze

Data from six participants of each group were excluded due to meeting exclusion criteria (see Measures section). No significant differences were found in initial rigidity scores between the mindfulness group (*M* = 2.78, *SD* = 2.09) and the waiting list group [*M* = 3.50, *SD* = 2.00; *t*(62) = 1.41, ns; Post hoc power = 0.40]. This lack of group differences in initial rigidity scores remained after additionally excluding participants who did not take part in the second experimental session [*t*(55) = 1.21, ns; Post hoc power = 0. 27], and participants who were excluded from the main analysis of the water-jar task [*t*(45) = 1.44, ns; Post hoc power = 0.33]. Thus, groups were statistically equivalent in initial rigidity scores.

#### Water-Jar Task

Four participants from each group chose not to participate in the second experimental session, and are therefore missing water-jar task data. Water jar data were excluded from two additional meditators who attended less than four program meetings. Nine participants (3 meditators) met water jar exclusion criteria (see Experiment 1). Two additional participants of each group were excluded from main water-jar analyses, one who reported being familiar with the task and its objective, and three others failed to properly comprehend jar usage instructions. Thus, data on main analyses are reported from 27 meditators and 26 controls.

In accordance with our hypothesis, following the mindfulness program, the mindfulness group (*M* = 2.19, *SD* = 2.09) received significantly lower rigidity scores than the waiting list group [*M* = 3.42, *SD* = 2.18; *t*(51) = 2.11, *p*<.02, 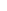 ^2^ = 0.08; Post hoc power = 0.67]. This effect slightly increased in a one way ANOVA in which Age and PET scores were added as Covariates to the analysis [*F*(1,49) = 6.28, *p*<.01, 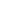 ^2^ = 0.11; Post hoc power = 0.81 ]. In the latter analysis, a main effect was found for PET scores, with higher rigidity scores attributed to those with low PET scores [*F*(1,49) = 5.76, *p*<.05], indicating that academic ability is negatively related to Einstellung rigidity. Group differences in rigidity scores remained significant in a similar ANOVA which included the nine participants who had met the water jar task exclusion criteria specified in Experiment 1, the participant who was familiar with the task, and the two participants who participated in less than four meetings [*F*(1,61) = 4.24, *p = *.02, 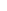 ^2^ = 0.07; Post hoc power = 0.70], verifying that the effect is not due to exclusion criteria. A series of t-tests comparing groups’ rigidity scores using various cutoff times for extinction trials (60,90,120 seconds) revealed that group differences remained significant on all cutoff times (minimal *t* = 1.88, *p* = .03, 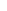 ^2^ = 0.07; Post hoc power = 0.63).

## Discussion

Mindfulness meditation has been described as involving adoption of a “beginner’s mind” and “being in the present moment” [31]. We therefore hypothesized that mindfulness may reduce cognitive rigidity and immune one from being “blinded” by past experience, as measured by the Einstellung water jar task. In Experiment 1, as hypothesized, following repetitive experience with a complex problem solving method, experienced mindfulness meditators were less blinded by experience and were better able than pre-meditators to identify the simple novel solution. In Experiment 2, similar results were obtained following mindfulness training in which participants were randomly assigned to mindfulness training vs. waiting list groups. These findings lend support to the notion that mindfulness involves cultivation of a “beginner’s mind”, and demonstrate that mindfulness practice reduces cognitive rigidity via the tendency to overlook simple novel solutions to a situation due to rigid and repetitive thought patterns formed through experience.

The present findings coincide with previous findings in which meditators outperformed non-meditators in tasks such as verbal fluency [52], [53], and visual perspective switching [51], in the respect of exhibiting an improved ability to generate varied responses to the same stimuli following mindfulness practice. Findings of the current study also coincide with previous findings indicating that meditators may exhibit decreased interference in the Stroop [49], [50] and Hayling [53] tasks in the sense of decreased automatic and habitual responding following mindfulness practice. Our findings additionally converge with findings regarding decreased rumination [38]–[40] in the sense of a reduction in repetitive and perseverative negative thoughts (see [63] for a discussion regarding such repetitive thinking). Findings of the current study bear novel contributions to the existing literature firstly by demonstrating that reductions in such rigid repetitive thinking patterns following mindfulness practice are evident regardless of thought valence or specific content and therefore reflect reduction in cognitive rigidity rather than a specific reduction in rigid ruminative content. Note that the measures of verbal fluency, visual perspective switching, Stroop, and Hayling tasks mentioned above, measure the ability to overcome over-learned habits rather than repetitive thinking patterns which have just been formed as does the Water jar task. An additional and central novel contribution of this study regards the increased ability to identify and utilize simple novel yet obvious solutions despite having experienced a successful, albeit complex approach in the recent past. Interestingly, the benefit of mindfulness was not restricted to years of experience and was found even following a six-week intervention.

Individuals suffering from depression and particularly those at suicidal risk tend to exhibit a narrowing of perceived options and difficulty in considering alternatives, a tendency which may prove fatal [64]. A similar narrowing of thought and difficulty in considering alternatives often occurs in instances of alcohol use and abuse. This phenomenon has been termed “alcohol myopia”, and has been found to facilitate extreme social responses [65], [66]. Although in need of future examination, it is tentatively suggested in light of the current results that reductions in such “blindness” to alternatives due to rigid thought patterns may (partly) underlie the efficacy of mindfulness in treatment of the above conditions [34], [41].

Additional implications of the current findings may regard other common situations, clinical as well as non-clinical, in which individuals may be rigidly “blind” to adaptive solutions or alternative courses of action due to previous experience. As illustrated in the beginning of this paper, overlooking the correct clinical diagnosis often occurs in cases that seem familiar but do not actually match clinicians’ past experience [1]. Mental health professionals often have difficulty in offering new perspectives and reframing the situation after having repeatedly heard their clients’ impressions [2], [3], and that managers and decision makers in organizations often experience trouble replacing existing procedures with more adaptive ones [4]. Findings of the current study suggest that mindfulness training may be useful in these and similar cases.

A number of methodological limitations should be considered regarding the present studies. Sample size, particularly in Experiment 1, was relatively small, due to the difficulty in reaching and recruiting the relevant populations. In Experiment 2, we compared a mindfulness intervention group with a waiting list group. This comparison enables the examination of the effects of a mindfulness intervention program as a whole rather than the effects of specific components within the intervention such as experiencing intervention. A third limitation concerns the water jar task. One of the main merits of this task is its examination of the tendency to overlook novel yet obvious solutions due to experience. This merit, however, also encompasses a shortcoming of the task, which is the fact that it may only be efficiently administered once. While this prevented examination of performance on the same task pre and post intervention, the equivalent performance on a task similar in structure at baseline level and the random assignment to groups practically resolves this shortcoming.

Future research may examine rigidity related effects that are specific to mindfulness interventions. This may be done by comparing mindfulness with active control groups and other intervention programs. A second direction for future research involves investigating the relationship between the effects of mindfulness training on the Einstellung effect and salient clinical outcomes in various forms of psychopathology. This may both extend the validity of the water jar task, a rigidity measure previously examined primarily in healthy individuals [6], to include rigidity in psychopathology, and may allow making more clearly established clinical implications than could have reached in the current study.
